# An Evidence-Based Digital Prevention Program to Improve Oral Health Literacy of People With a Migration Background: Intervention Mapping Approach

**DOI:** 10.2196/36815

**Published:** 2023-05-11

**Authors:** Marie-Theres Weil, Kristin Spinler, Berit Lieske, Demet Dingoyan, Carolin Walther, Guido Heydecke, Christopher Kofahl, Ghazal Aarabi

**Affiliations:** 1 Department of Periodontics, Preventive and Restorative Dentistry Center for Dental and Oral Medicine University Medical Center Hamburg-Eppendorf Hamburg Germany; 2 Department of Prosthetic Dentistry Center for Dental and Oral Medicine University Medical Center Hamburg-Eppendorf Hamburg Germany; 3 Department of Medical Sociology Center for Psychosocial Medicine University Medical Center Hamburg-Eppendorf Hamburg Germany

**Keywords:** oral health, health behavior, oral health knowledge, migration, mobile health, mHealth, preventive dentistry, intervention mapping, mobile phone

## Abstract

**Background:**

Studies in Germany have shown that susceptible groups, such as people with a migration background, have poorer oral health than the majority of the population. Limited oral health literacy (OHL) appears to be an important factor that affects the oral health of these groups. To increase OHL and to promote prevention-oriented oral health behavior, we developed an evidence-based prevention program in the form of an app for smartphones or tablets, the Förderung der Mundgesundheitskompetenz und Mundgesundheit von Menschen mit Migrationshintergrund (MuMi) app.

**Objective:**

This study aims to describe the development process of the MuMi app.

**Methods:**

For the description and analysis of the systematic development process of the MuMi app, we used the intervention mapping approach. The approach was implemented in 6 steps: needs assessment, formulation of intervention goals, selection of evidence-based methods and practical strategies for behavior change, planning and designing the intervention, planning the implementation and delivery of the intervention, and planning the evaluation.

**Results:**

On the basis of our literature search, expert interviews, and a focus group with the target population, we identified limited knowledge of behavioral risk factors or proper oral hygiene procedures, limited proficiency of the German language, and differing health care socialization as the main barriers to good oral health. Afterward, we selected modifiable determinants of oral health behavior that were in line with behavior change theories. On this basis, performance objectives and change objectives for the relevant population at risk were formalized. Appropriate behavior change techniques to achieve the program objectives, such as the provision of health information, encouragement of self-control and self-monitoring, and sending reminders, were identified. Subsequently, these were translated into practical strategies, such as multiple-choice quizzes or videos. The resulting program, the MuMi app, is available in the Apple app store and Android app store. The effectiveness of the app was evaluated in the MuMi intervention study. The analyses showed that users of the MuMi app had a substantial increase in their OHL and improved oral hygiene (as measured by clinical parameters) after 6 months compared with the control group.

**Conclusions:**

The intervention mapping approach provided a transparent, structured, and evidence-based process for the development of our prevention program. It allowed us to identify the most appropriate and effective techniques to initiate behavior change in the target population. The MuMi app takes into account the cultural and specific determinants of people with a migration background in Germany. To our knowledge, it is the first evidence-based app that addresses OHL among people with a migration background.

## Introduction

### Background

Previous research has demonstrated poorer oral health among many migrant populations compared with the majority of the population of the respective country [1-3]. Individuals with a migration background often exhibit different oral health behaviors and regularly encounter barriers to accessing preventive dental care [4-7]. Oral health literacy (OHL) is defined as the ability to obtain, understand, and process information to make appropriate and reasonable decisions regarding one’s own oral health [8,9]. Hence, OHL is an important factor that affects health behavior and subsequently health outcomes. For example, studies demonstrate an association between limited OHL and a higher prevalence of severe periodontitis [9,10]. Some studies revealed that people with a migration background score lower than the majority of the population on certain components of OHL, for example, the ability to comprehend oral health knowledge [11,12]. Thus, it seems plausible that a lower OHL could be decisive for the oral health differences between people with and without a migration background in Germany. Testing this hypothesis was one of the aims of the Förderung der Mundgesundheitskompetenz und Mundgesundheit von Menschen mit Migrationshintergrund (MuMi; promotion of OHL and oral health of people with a migration background) research project, which was conducted at the University Medical Center Hamburg-Eppendorf [13] between July 2018 and June 2022.

Tailored health education is regarded as an essential tool for improving health literacy [14]. Central to such health education is the targeting of health behavior. This emphasizes the need for interventions that improve oral health knowledge and focus on changing oral health behavior. Owing to the increased prevalence of smartphones and awareness among health care professionals for the potential of digital interventions to target heterogeneous populations as well as specific needs, the field of mobile health (mHealth) is growing vastly [15,16]. Therefore, mHealth apps may be an effective delivery method for providing health education. Currently, >100,000 apps are available in the categories *Health and Fitness* and *Medicine* [17], and 75% of smartphone owners used health apps in 2020 [18]. These apps have great potential to provide low-threshold access to information and preventive measures [19].

### Objectives

Given the lack of well-rounded evidence-based approaches to oral health prevention, we decided to develop the MuMi app, which aims to improve the OHL and subsequently the oral health of its users, especially susceptible groups, to contribute to oral health equality in Germany. Thus, the second aim of the MuMi project was to examine the appropriateness and effectiveness of the MuMi app for improving OHL and consequently the oral health of people with a migration background. The development, production, implementation, and evaluation of the MuMi app took place within the MuMi project.

To ensure that the app is evidence-based and feasible to use for the targeted population, we implemented the intervention mapping approach (IMA). This paper provides a detailed description and analysis of the systematic development of the MuMi app in terms of its content and structure.

## Methods

### Description of the IMA

The IMA is a planning protocol for theory- and evidence-based behavior change intervention programs [20-22]. It has been used for health promotion purposes and program implementation strategies [23]. In the field of health education, the scientific focus is often on effectiveness rather than the actual development of such programs [22]. Therefore, the IMA provides guidance on how to identify theory-based determinants of the targeted behavior and apply behavior change methods into a practical evidence-based health promotion program to reach the desired health outcomes [22,24]. Owing to the detailed steps, the approach is time consuming, but it ensures that (1) the development process of the intervention is carried out in a systematic, evidence-based, and transparent manner, which is also comprehensible for outsiders, and (2) that the intervention is implemented as planned. The IMA has been applied across various settings, fields, and programs, including digital interventions [20,25,26].

**Figure 1:**
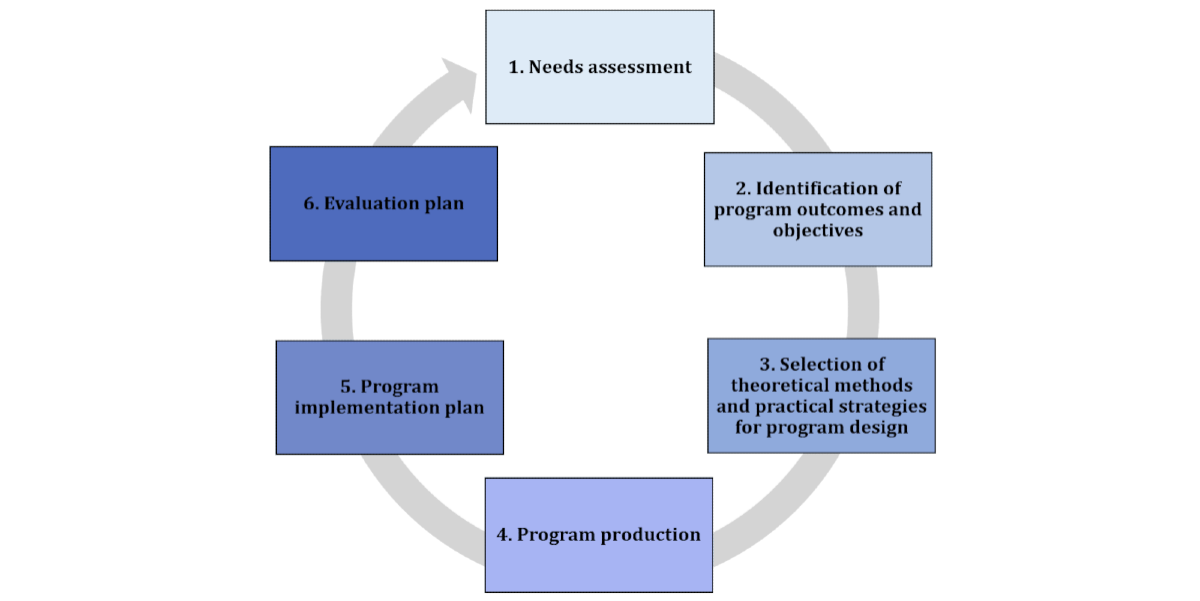
The 6 steps of the intervention mapping approach.

IMA is a participatory approach that involves the target population, program implementers, and community and stakeholder engagement. In addition, the IMA guides the use of theories and evidence to better understand the behavioral causes of health problems, which may be influenced by the structural conditions of the environment, and to identify their determinants to select appropriate behavior change methods. Finally, the IMA follows a social-ecological perspective, which recognizes that social, physical, and environmental conditions influence the behavior of the individual [27]. Overall, the implementation of the IMA is divided into 6 consecutive steps (Figure 1).

The development of the intervention was guided by the MuMi project team, consisting of 3 dentists with research experience, 1 psychologist with expertise in health care research, 1 psychologist with expertise in migration research, 1 molecular biologist, and 2 health scientists. One-third of the project team had a migration background, and their specific experiences were incorporated into app development as well.

### Step 1: Needs Assessment

The first step of the IMA is an extensive needs assessment and problem analysis of the target group. This involves determining the initial situation of the target population, identifying their needs, and thus defining the relevant problems and specific health-related behaviors as well as their causes.

First, the multidisciplinary team conducted an extensive search of the literature in various databases such as PubMed. We specifically focused on studies identifying the oral health and OHL of people with a migration background in Germany.

Second, to capture a broad spectrum of opinions and perspectives, expert interviews and a focus group were conducted with the target population. All qualitative data were analyzed using thematic analysis. The experts were selected according to a gatekeeper strategy, that is, people who had an extensive overview of and access to potential experts were asked to name such experts and invite them to participate in the interview [28]. The MuMi project team identified and contacted 6 suitable interviewees. In a snowballing process, 3 more experts were identified and contacted via the first interviews. The number of expert interviews was deemed sufficient once a representative of every relevant field (dentistry, migration research, health research, and psychology) was interviewed. In total, 9 expert interviews were conducted with people with strong vocational interactions and experiences within the appropriate sector.

The interviews followed a semistructured interview guide and were conducted either via telephone or face-to-face. At the beginning, the experts were given a brief introduction of the background and aim of the interview. They were then asked to speak freely about their professional experiences and impressions of the topic of *migration and oral health*. Then, they presented their knowledge and experiences with the dental care utilization of people with a migration background, their impressions of differences in oral health owing to cultural influences and diversity, and crucial cultural factors for motivating people with a migration background regarding their oral health care. Finally, they were asked to describe their ideas and suggestions for better oral health care and possible preventive measures for people with a migration background.

Focus group participants were recruited according to the principle of theoretical sampling [29]. Participants were recruited from different dental practices. According to the snowball method, additional participants were recruited via the participants recruited in the dental practices. The inclusion criteria were migration background, aged at least 18 years, proficiency in German or Turkish, and living in Germany. Participants were contacted personally and received written information about the content and procedure of the interview. The focus group consisted of 7 participants. The discussion was led by 2 team members using a semistructured guideline. All participants were of Turkish origin, as they are the biggest migrant community in Germany [30]. Questions for the interviewees were regarding the German dental health care system, access to services, and cultural determinants. All the participants provided informed consent.

All key themes and statements that emerged during the group discussion were used to develop a content mind map. Once the overlapping information provided by the study participants was substantial and no new themes emerged, content saturation was reached, as agreed by all participants. All interviews were recorded and transcribed. Qualitative data analysis was performed using MAXQDA software (VERBI Software) and analyzed according to the analysis recommendations by Creswell and Poth [31]. Key themes were generated deductively, based on prior research, and inductively, based on the content. For more information, the results of the expert and focus group interviews, including recruitment of participants, data collection, and analysis, were published in a separate publication [32]. On the basis of the results of the literature research, expert interviews, and focus group, the project team selected changeable determinants of oral health behavior that could be achieved by the intervention.

### Step 2: Identification of Program Outcomes and Objectives

Step 2 involves the identification of program objectives and outcomes that indicate unhealthy behaviors to achieve the overall aim of the program, that is, to improve OHL and oral health outcomes in people with a migration background. The performance objectives (POs; the “what to do”) for the relevant population at risk formalize the change objectives (COs; the “why to do”) that are needed to achieve the program outcomes. The COs include the determinants of POs such as awareness and knowledge, risk perception, access to health care, self-efficacy, attitudes, and social norms and support. These must be identified, assessed, and addressed to achieve changes in POs. In other words, COs are defined as a combination of POs and their modifiable determinants.

As originally suggested by Bartholomew in 1998 [22], the project team constructed a matrix listing the relevant POs, their determinants, and the COs derived from them. However, creating a matrix that considered all health behaviors as intended proved to be quite complex and would have resulted in an overwhelming amount of information. Owing to time constraints imposed by the project’s milestone planning, the creation of the matrices, therefore, had to focus on the most relevant POs and determinants, as suggested by the interviewees and focus group members who had any potential for change (eg, knowledge can be changed more or less easily, yet individual socialization cannot). The dentists, psychologists, and health scientists of the research team agreed on the most relevant POs and determinants through mutual discussion.

### Step 3: Selecting Theoretical Methods and Practical Strategies for Program Design

In the third step, we first searched for suitable theoretical methods that have been proven to enable change in the identified behavioral determinants. We chose theoretical methods on the basis of existing oral health promotion interventions [33-37]. Afterward, we tailored the theoretical method to the setting and target population (a practical strategy). Opinions from experts from the mHealth industry were sought to ensure the appropriate selection of practical strategies for implementation in a smartphone app.

### Step 4: Program Production

Step 4 of the IMA focuses on the actual design of the program. The structure, design, materials, utilities, and functions were created and produced. Together with an app development company, a graphic designer, and a translation agency, the project team combined and clustered the chosen methods and strategies of step 3 into a program plan. Changes or suggestions for improvements to optimize the program plan were discussed as part of regular exchanges between all those involved in program production. To ensure that the app meets the target population’s needs and to check the app against pitfalls, errors, and shortcomings, a test phase of the beta-version was conducted. After this first test, the system errors, content, and technical aspects were optimized. For the following intensive pilot testing, 20 people (10 with a migration background and 10 without a migration background) were recruited in dental practices to test the app. In addition to migration status, age, sex, and education were collected from all pilot testers to ensure representation of different age groups, both sexes, and people of different socioeconomic status (SES). Test users were asked to provide feedback on (1) language, for example, was something not understandable or were there linguistic comprehension difficulties; (2) graphical user interface, for example, visualization of icons, images, and audio; (3) functionalities, for example, user-friendliness or suggestions for improvements of functions; and (4) bugs, for example, were there problems or error reports when using the app. Detailed feedback was sent via email to the project team and forwarded to the app developers for improvement.

### Step 5: Program Implementation Plan

For the penultimate step, we determined how the realization and implementation of the program will be carried out, that is, in which setting and in which target group. Collaboration with future program implementers from the beginning was regarded as extremely important to ensure successful implementation. The development of the MuMi app took place within a randomized controlled trial (RCT) with dental practices offering the MuMi app to their patients (intervention group) and practices that do not (control group). The involvement of dental health professionals was done for the reason of providing the app to their patients, to increase the dentists’ acceptability of the app, and to follow the study protocol with its documentation and examination necessities.

### Step 6: Evaluation Step

The last step involves the evaluation of the implemented program to assess its effectiveness and the implementation process. In our case, the MuMi app was evaluated within the MuMi project. Here, we only present a brief overview of the evaluation of the intervention. Comparisons between app users and the control group were performed with respect to sociodemographic variables, OHL, and clinical parameters.

### Ethics Approval and Informed Consent

All procedures performed in the MuMi project were approved by the ethics committee of the Universitätsklinikum Hamburg-Eppendorf (local psychological ethics committee at the Center for Psychosocial Medicine; LPEK-0027). For the focus group discussion, all participants provided informed consent, and ethics approval for the focus group was given by the medical association Hamburg (number: PV5827). Written informed consent and privacy statements were obtained from all participants before participation and use of the MuMi app. The collected data were processed and evaluated pseudoanonymously.

## Results

### Step 1: Needs Assessment

#### Task 1.1: Literature Search

To better understand the reasons for differing OHL and oral health status, health behavior, and utilization patterns, we conducted a systematic literature search on the quantitative evidence regarding the OHL of migrants and ethnic minorities [38]. Subsequently, the literature on the oral health of people with a migration background in Germany was screened. In 2019, approximately one-quarter (26%) of Germany’s population had a migration background, defined as people who have immigrated or have at least 1 parent not born as a German citizen [39]. The literature search revealed considerably poorer oral health among several groups with a migration background compared with the majority of the population in Germany [40,41]. Most migrants from non-Western countries tended to have predominantly problem-oriented dental care utilization patterns and hence made less use of preventive services, such as checkups and professional dental cleaning [42]. Moreover, the target population largely showed a lower tooth brushing frequency and use of drugs for caries prophylaxis [43,44]. SES was identified as another risk factor for poor dental health [45]. Although migrant status is strongly associated with SES [46], SES is not the only explanation; migration background itself remains an independent and discrete predictor of poor oral health [47]. This suggests that certain migration- and culture-specific factors may affect the oral health of a person.

#### Task 2.1: Expert and Focus Group Interviews

During the expert and focus group interviews, language barriers, which hinder patient-doctor communication and the understanding of health information, were identified as the main obstacle. Deficits in knowledge about the German health care system and oral health in general as well as its importance for the overall health of an individual were also identified as major barriers. A member of the German dental board explained the following [32]:

(Among migrants) there was only little knowledge about oral health measures. With respect for the different backgrounds certainly understandable; but the problem of caries as a health threat – as it is socialized in Germany –, is not established – that one considers it as important to reduce this disease by the improvement of their own oral hygiene.

Furthermore, health socialization also seemed to play a major role. People who grew up in different social and health care systems had learned different health-related behaviors and were accustomed to the rules and regulations of a foreign health care system rather than the German one. Experts and the target population also reported that health practices were influenced by the perceived value and significance of oral health, which in turn depends on the level of knowledge, beliefs, and structure of the health care system. One participant in the focus group expressed the following [32]:

One has little time for things that are important. And it is not important to visit the dentist when one has no ailment.

Among other things, these factors might lead to largely problem-oriented dental care utilization patterns, as identified by the data of previous studies identified in our literature search. Furthermore, several culture-specific eating and oral hygiene habits were found to be important factors in determining the oral health of people with a migration background, such as the sweetening of tea with honey, specifically in Arab countries, or regularly offering sweets to guests. The detailed results of the expert and focus group interviews have been published elsewhere [32].

In summary, language barriers, lack of oral health knowledge, and low significance of oral health were identified as the main factors that influence risk behavior, such as problem-oriented utilization patterns, inefficient oral hygiene practices, and teeth-unfriendly nutrition patterns. A migration background, lower SES, health socialization, and differing health care systems (environmental conditions) contribute to health problems. On the basis of these results and the literature on behavioral determinants that best explain these behaviors, the project team selected the most important modifiable determinants [27,48,49]. “Knowledge” was identified as an essential determinant for all POs.

### Step 2: Identification of Program Outcomes and Objectives

The program outcomes of the MuMi project were as follows: (1) improve OHL and subsequently contribute to behavioral change among the app users and, through behavioral change, (2) to lead to better oral health outcomes. In combination with the results of step 1 and appropriate behavioral change theories—such as the health action process approach [50], which includes the transformation of behavioral intentions into actual behavior, thus filling the gap between intentions and behavior—we then specified the POs.

Using the final determinants to target, 4 POs for behavioral outcomes were defined (Multimedia Appendix 1). Once the POs were specified, we created the COs by linking the POs to behavioral determinants. For example, if the PO was “App-users establish an efficient oral hygiene,” appropriate behavioral determinants would be “risk perception,” “awareness,” “knowledge,” “self-efficacy,” and “skills.” “Risk perception” is responsible for the person to be aware of the risks of dental diseases and their own susceptibility to them and to be aware of the impact of inefficient oral hygiene practices on oral health or overall health. In addition, the person must be aware of their own oral health (“awareness”) and know what good oral health behavior is and what techniques (eg, tooth brushing) to adopt (“knowledge”). “Self-efficacy” is responsible for the person to feel confident and positive about their oral health behaviors. Finally, the person must use appropriate oral hygiene products (“skills”). In total, 4 POs and 20 COs were defined (Multimedia Appendix 1).

### Step 3: Selecting Theoretical Methods and Practical Strategies for Program Design

#### Overview

During the development process of the MuMi app, methods and strategies were sought to achieve the planned intervention goals, which included the provision of risk information, video-based education, cues and reminder systems, and incentives for self-control and monitoring.

**Figure 2:**
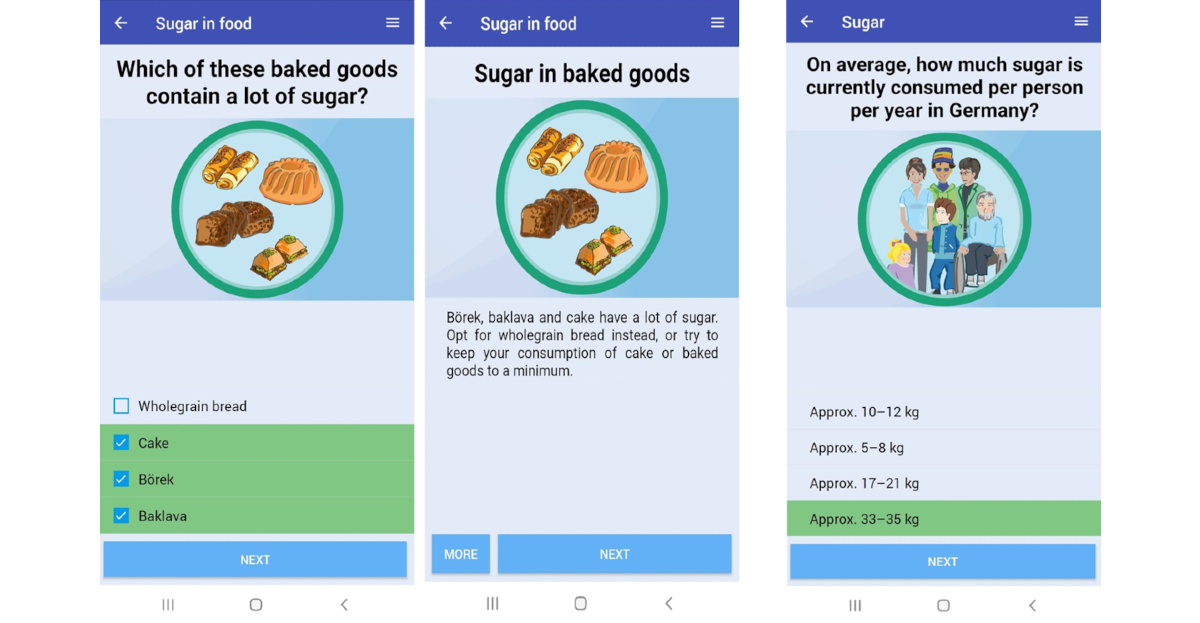
Screenshots of the example questions of the knowledge quiz with explanatory text.

To achieve POs 2 to 4 in Multimedia Appendix 1, the predominant theoretical approach within the MuMi app was the *provision of information* about oral health, specifically concerning behavior-related risk factors [33,51]. The literature supports the effectiveness of educational interventions on health-related behaviors and outcomes [35,52]. To engage the user, the information was delivered using another theoretical approach called *entertainment education* [51]. This method is defined as providing a form of entertainment designed to educate about health behavior as well as to entertain. Important parameters for effectiveness, that go with this method, are the consideration of source and channel and the balance of media professionals’ and health promoters’ needs [51]. Thus, we first put together a collection of evidence-based information about oral health and then sat down with the app developers to determine how to deliver this information to our participants in a way that was entertaining and not too complex. Together, we decided to create the app as a game in the form of a quiz with multiple-choice questions, enriched with explanatory and instructional texts (Figure 2). The app developers gave us a structure in the form of Microsoft Excel spreadsheets, in which we could compile the information. We developed different questions with 4 different answers, in which one or more answer options could be considered correct. We specified in which cases a question or statement should be followed by an explanatory article or which pictograms and videos should be added. We also defined the thematic layout and the order of the app.

Although imparting knowledge is essential for health literacy change, oral hygiene instructions are often not sufficient to affect changes in oral health behavior [53]. Instructions accompanied by written and visual illustrations were considered more successful. Therefore, another theoretical approach to achieving the intervention goal of establishing efficient oral hygiene was *video-based education* [54]*.* In practice, it has been implemented in the form of videos, such as a demonstration of a proper tooth brushing technique or the use of dental floss. Anatomically correct depictions of the teeth and periodontium were provided in the illustrations. The use of *cues and reminder systems* has also been identified as a theoretical method for changing behavior [51,55]. The literature supports that sending reminder text messages can help improve oral health behavior and oral health knowledge [56]. Therefore, the sending of so-called push notifications at predefined time intervals was programmed as an additional function in the MuMi app. These notifications remind the user to brush their teeth twice a day, use oral hygiene aids (eg, interdental brushes), change their toothbrush, go for their next dental appointment, or gain more knowledge about oral health by using the MuMi app. For *self-control and monitoring*, a behavior change method that was frequently mentioned in the literature, the so-called progress bar [37,51,55], was included to allow users to track their progress in the app (How much did they already learn? Which answers were right or wrong?). It was stipulated that users’ motivation was to be increased by tracking the progress bar [57].

#### Cultural Sensitivity

To overcome language barriers and to target different subgroups of the migrant populations from different countries, the app was made available in 5 languages: German, English, Arabic, Turkish, and Russian. The project team ensured to use simple language with little dental jargon. Considering the cultural and ethnic diversity of the target population, we included illustrations of a diverse group of people, for example, different ethnicities, sexes, and age groups, to communicate oral health–related information so that various subgroups of the target population could be addressed. Furthermore, with regard to nutrition and dietary habits, examples of food items from different countries were provided, for example, Baklava, a typical pastry from Turkey, and dried fruits or honey, which are often consumed in Middle Eastern countries. Both contain high amount of sugar. Thus, instead of using only examples of sugary foods that are typical in Germany, such as gummy bears, these examples were incorporated into multiple-choice questions and illustrations. Finally, links with additional information on where to find interpreters or what medical services an individual is entitled to receive, depending on the residency status, were included in the app.

### Step 4: Program Production

#### Overview

The next step of the IMA was the planning and design of the intervention, in which the structure and functions of the MuMi app were defined. For this purpose, the literature was screened to fill the instructional content of the MuMi app with evidence-based information. The guidelines for the prevention of caries and periodontitis by the Association of the Scientific Medical Societies in Germany were found to be the best available (gold standard) [58-61]. In addition, dental specialist literature was included [62,63], and publications concerning particular dental topics were added [64-66]. For the technical implementation (Which content can be implemented? and How should the structure of the app be designed?), the opinions of experts from the mHealth industry were sought throughout the production cycle.

The content of the MuMi app was structured into three main pillars: (1) What should I do? (2) What should I know? and (3) Who can help me? First, after collecting the relevant information, we created mind maps to organize the relevant information and to form relationships among the pieces. On this basis, we formed 10 content modules assigned to the 3 pillars (Table 1).

The first pillar (“What should I do?”) covers the topics related to oral hygiene, nutrition and fluorides, and dental health care utilization. For the second pillar (“What should I know?”), information about oral diseases, risk factors, and anatomy is presented. The third pillar (“Who can help me?”) includes content on topics related to the health care system in Germany as well as the possible sources for dental information and general information related to what happens in dental practices based on the directives and regulations of the German health care system. The creation of this informational content was carried out by a multidisciplinary team, and it was reviewed and adapted several times in terms of evidence, correctness, completeness, and comprehensibility.

In cooperation with the app development company, content tables for the 3 pillars with correct and other possible answer options for the multiple-choice quiz were created until they were optimized through continuous feedback loops between the project team and the app developers. Content tables for the cues and reminders, for example, to brush teeth twice a day, were also created and shared, including the text of the push notification that is supposed to appear on the user’s phone, as well as how often the user should receive the push notifications, for example, once per week. All pictograms and videos that illustrate the content were designed by a graphic designer in cooperation with the dentists of the MuMi team to ensure realistic and anatomically correct depictions. The entire software programing was done by the app development company.

**Table 1:** Content overview of the Förderung der Mundgesundheitskompetenz und Mundgesundheit von Menschen mit Migrationshintergrund (MuMi) app.

Pillars and modules			Content
**Pillar 1: behavior (what should I do?)**			
	Diet and nutrition	Acidity (food items, amount of intake, and consequences) Sugars (food items, amount of intake, and consequences)	
	Oral hygiene	Toothbrushing (techniques, frequency, duration, and what type) Toothpaste (what type, quantity, and ingredients) Floss, interdental brush, and mouth wash (techniques, frequency, duration, and what type) Individual prophylaxis and professional dental cleaning	
	Fluoride	Toothpaste Mouth wash Foods Fluoride gel Food items and drinks	
**Pillar 2: knowledge (what should I know?)**			
	Basics	Caries Periodontitis Dental plaque Highly concentrated fluoride varnish Fissure sealing Impact of dental health on overall health and quality of life	
	Risk factors	Smoking Diabetes Age Gender SES^a^ Stress	
	Anatomy	Structure of teeth Oral mucosa Mandibula joint Masticatory muscle Soft tissue	
**Pillar 3: health care system (who can help me?)**			
	Health care system	Structure of the health care system Financing Behavioral patterns	
	Dentist visit	How to find a dentist What to prepare for a dentist visit What examinations are carried out during a checkup	
	Dentistry specializations	Prosthetic dentistry Oral and maxillofacial surgery Orthodontics	
	Sources of information	Internet Official guidelines Dental offices	

^a^SES: socioeconomic status.

#### Translation of the MuMi App

**Figure 3:**
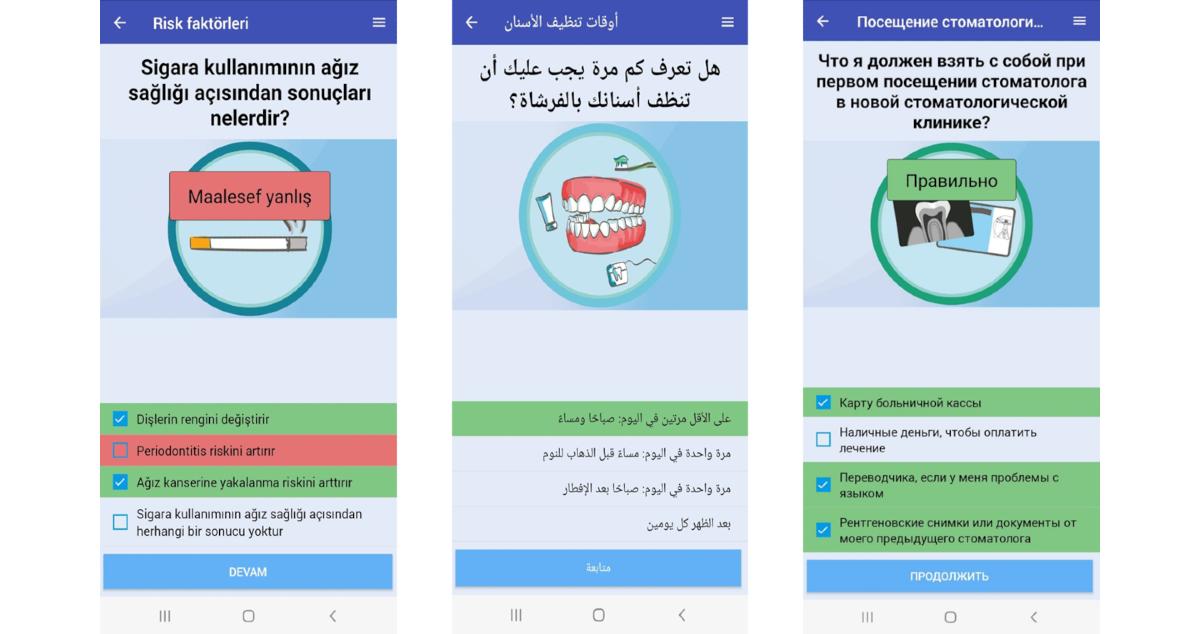
Screenshots of the Förderung der Mundgesundheitskompetenz und Mundgesundheit von Menschen mit Migrationshintergrund (MuMi) app knowledge quiz in Arabic, Russian, and Turkish.

The MuMi app was translated into English, Arabic, Turkish, and Russian according to a multistep team approach by Harkness [67]. All translations were performed using forward or backward loops between the project team and the professional translators. The initial translation was performed by bilingual translators with translation experience from a translation agency. Afterward, professional translators reviewed the initial translations. On the basis of the feedback from the professional translators, all texts were revised by the initial translators. Subsequently, all texts were reviewed by bilingual project staff and revised if necessary. Figure 3 gives an example of MuMi app multiple-choice questions and explanatory text in Arabic, Russian, and Turkish.

#### Pilot Test of the MuMi App

All app features were tested after each programming cycle, as described in *Step 2: Identification of Program Outcomes and Objectives* subsection under the *Methods* section. The test participants were very motivated and reported accurate; comprehensive; and detailed errors, bugs, ambiguous wording, and so on.

#### Final Program: The MuMi App

**Figure 4:**
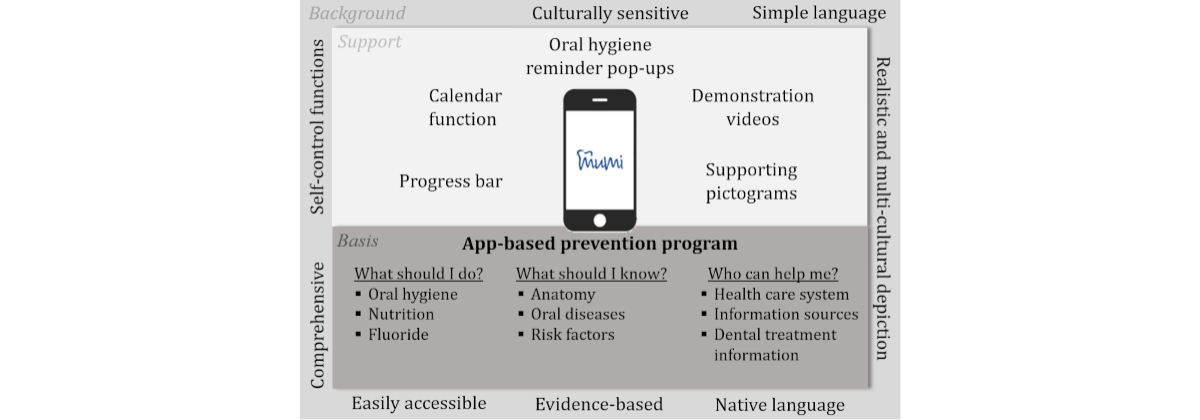
Förderung der Mundgesundheitskompetenz und Mundgesundheit von Menschen mit Migrationshintergrund (MuMi) app functions and content.

At its final stage, the MuMi app has >350 different questions and educational elements that are organized into 3 main categories. The app’s core features are an activity screen with a multiple-choice quiz and provision of information on the topic, self-monitoring and progress bars, and reminder notifications. Figure 4 provides an overview of the app functions and contents.

The app is available for iPhones, iPads, and Android systems. Potential users can download the app from the Android app store or the Apple app store (important note: log in as *guest*, not via QR code which was applicable only for study participants).

### Step 5: Program Implementation Plan

The development and implementation of the MuMi app took place within the MuMi project. The intervention was planned to be delivered by dental professionals working in dental practices. The dental practices were chosen based on their location, with a focus on a high degree of population diversity and people with a migration background among their patients [68]. In total, 40 dental practices agreed to take part in the MuMi project. All cooperating dental practices signed a cooperation agreement. The dental surgeries were clustered and half of them were randomized to be part of the intervention group, which had to offer their patients the MuMi app. Before the recruitment of patients, the project team presented the MuMi app and the project implementation plan to the dental surgery staff through presentations and practical exercises in a full-day workshop at the university hospital.

### Step 6: Program Evaluation

Study participants were of legal age (≥18 years), possessed a smartphone or tablet, and were recruited by the dental surgery staff. Participants were identified as having a migration background if they themselves or at least 1 parent was not born in Germany. Patients in the intervention group were invited to test the MuMi app over a period of 6 months. They received a QR code to scan when downloading and registering for the app. The code was assigned to the relevant dental surgery for subsequent data analysis. The control group received usual care. The OHL and clinical oral health of all participants in the intervention and control groups were assessed and documented before app use (baseline) and after use of the app (6 months follow-up). Clinical oral health was assessed using the following clinical parameters: dental status (number of decayed, missing, and filled teeth), approximal plaque index (API), and sulcus bleeding index. Practicing dentists of the recruited dental practices were trained by the MuMi project team on how to conduct clinical assessments. OHL was measured using the Oral Health Literacy Profile, an instrument developed within the MuMi project [69]. In addition, participants in the intervention group were questioned about the feasibility and acceptability of the app as well as their app use behavior and how they experienced and assessed the app. The participating patients were given an oral hygiene kit as an incentive (toothbrushes, mouth rinse, dental floss, etc). Dental practices received a financial incentive of EUR €50 (US $1.09) per case.

The analyses of the total cohort showed that users of the MuMi app had a substantial increase in their OHL after 6 months compared with the control group. We also observed an association between improved API scores and app use in terms of oral hygiene (measured using API). An interaction term of app use and migration background showed improvements in OHL, specifically in migrants. This suggests that particularly susceptible groups, such as people with lower SES or people with a migration background, can benefit from the content of the app. Detailed results of the evaluation of the MuMi app will be published in the near future.

## Discussion

This paper describes the development process of the MuMi app, a digital oral health promotion and prevention tool to improve users’ OHL and subsequently their oral health.

### Comparison With Prior Work

Existing nonmedical oral health apps illustrate prosthetic or implantological treatments to patients and reference books or guides about dental treatments and medications [70,71]. The most commonly used oral health apps encourage good oral hygiene, for example, through toothbrushing timers or toothbrushing apps for children [70,72]. A theoretical basis and the use of behavioral change methods increase the effectiveness of an intervention [73]. However, many existing health apps for oral health promotion lack a theoretical foundation for their content and evaluation of their effectiveness and are not empirically validated [17,72]. Furthermore, the integration of behavior change methods is often limited in these apps. Considering the paucity of evidence-based apps, the MuMi app, on the contrary, has a sound theoretical basis with a comprehensive, systematic, and thorough development process.

Participation of the intervention group during the development process is known to be an integral component for intervention planning and participatory health research [22,74]. By applying the IMA, it is possible to focus on the target group and the intervention setting at every stage of the development process. The success of participatory health research in developing prevention and health promotion programs for susceptible groups has already been demonstrated in a project to prevent HIV infection among migrants in Germany [75]. Before starting the development of the MuMi app, we conducted focus group interviews to identify the main access barriers to dental treatment for the target population and to identify important culture- and migration-specific factors that had to be taken into account and integrated during the development process. The multidisciplinary team ensured the integration of the identified culture- and migration-sensitive content during all stages of the app development process. However, it is important to note that all the participants in the focus group were of Turkish origin. Although people with a Turkish migration background make up the largest group within the migrant population in Germany at 12.3% [76], the culture- and migration-specific characteristics of other countries and regions might differ and could therefore not be portrayed in the app. Viewing the target group as a collective does not do adequate justice to the diversity of individual societal and cultural backgrounds. In general, it can be assumed that there is a very high degree of heterogeneity among people with a migration background. These factors must be taken into account during the evaluation of the intervention. Nevertheless, our literature search revealed that language barriers and knowledge gaps were the most common barriers to accessing dental services among migrant populations of different origins.

Following all steps of the IMA carefully ensured that the oral health–related content as well as the entire development process of the MuMi app is based on evidence. The IMA has successfully been used in the development of other interventions [77-84]. In contrast, the literature on the use of the IMA for the development of mHealth interventions is less extensive. A Dutch team from the University of Amsterdam developed a mobile app, the WhiteTeeth app, which aims to promote good oral health behavior among Dutch adolescents with fixed orthodontic appliances [85]. The effectiveness of the app in improving oral hygiene was tested in an RCT [86]. The use of fluoride mouth rinse increased after the 6-week follow-up among the intervention group, and at the 12-week follow-up, the presence of plaque was reduced substantially more among the intervention group than the controls [86]. Overall, the IMA proved to be a very effective tool for developing an app for preventing dental caries, which was proven to help adolescents with fixed orthodontic appliances perform their oral self-care behavior at home. The effectiveness of the MuMi app was also tested in an RCT. OHL and API scores (indicator of oral hygiene) increased substantially after 6 months among app users in comparison with the control group. Additional studies that outlined the use of intervention mapping for developing mHealth interventions focused solely on steps 1 to 3 [87] or steps 1 to 4 [88,89]. In that sense, the MuMi app differentiates itself from other apps using the IMA that have not been tested in a trial.

In recent years, the German Federal Ministry of Health has drafted various laws, such as the Digital Care Act, which came into force in 2019 [90], to impart political impetus to the digitization of the health care sector. Among other things, its purpose is to provide physicians with a legal basis for prescribing digital health apps, and it acknowledges them as therapeutic instruments [90]. Thus, it would be possible for practicing dentists to prescribe the MuMi app as a tool for effectively improving oral health among susceptible populations.

To our knowledge, the MuMi app is the first evidence-based mHealth app that addresses OHL among migrants. We believe that this makes it a unique and promising mHealth program for OHL promotion. Although the app aims to target susceptible groups such as people with a migration background, its content addresses people regardless of their origin and cultural background. Thus, the app intends to be valuable for everyone who has rather limited access to oral health information.

In most published intervention effect–focused studies, the theoretical-based development process of an intervention is rarely addressed. However, it is precisely these development processes that should be available to other program planners, so that effective programs can be replicated by other researchers and further evidence-based development of health promotion interventions can take place [73].

### Limitations

This study has some limitations that should be addressed. Although we experienced that the IMA provided a suitable tool for the development process, it was very time consuming. Other researchers have similar experiences in this regard [85,91,92]. The health care–related content of the MuMi app is specific to the German health care system. Therefore, it is not suitable for use in other health care settings. However, the intervention can be easily adapted to other contexts and health care systems. Overall, the evidence-based information on tooth-friendly diets, preventive health behavior, and oral health knowledge can be universally implemented in all settings.

### Conclusions

The IMA was used to allow the MuMi team to create an evidence-based health intervention guided by a clear, structured plan, which puts the theories of health science into practice. The MuMi app is a novel and innovative app that aims to improve OHL and subsequently oral health. To make the app available to a wider range of people, the app could easily be translated into other languages or be further developed for use by children.

## Appendix

Multimedia Appendix 1 Implementation of the performance objectives of the Förderung der Mundgesundheitskompetenz und Mundgesundheit von Menschen mit Migrationshintergrund (MuMi) app.

## Abbreviations

API: approximal plaque index
CO: change objective
IMA: intervention mapping approach
mHealth: mobile health
MuMi: Förderung der Mundgesundheitskompetenz und Mundgesundheit von Menschen mit Migrationshintergrund
OHL: oral health literacy
PO: performance objective
RCT: randomized controlled trial
SES: socioeconomic status
