# Supplementary material for: An Evidence-Based Digital Prevention Program to Improve Oral Health Literacy of People With a Migration Background: Intervention Mapping Approach
Source: JMIR Form Res. 2023 May 11;7:e36815. doi: 10.2196/36815 (PMC10214121; doi:10.2196/36815)
Supplement: Multimedia Appendix 1 [file formative_v7i1e36815_app1.docx]

| Steps | Performance objectives | Change objectives | Implementation in the MuMi app |
| --- | --- | --- | --- |
| 1 | Understanding: app users overcome language and communication barriers | - Understand oral health–related information (“awareness” and “knowledge”) - Gain confidence to deal with communication barriers (“self-efficacy”) - Know how and where to find preventive services (“knowledge” and “coping”) - Receive support even without adequate German language skills (“support”) - Reduce dental anxiety caused by communication barriers (“self-efficacy”) - Learn what tools to use to overcome language barriers (“self-efficacy”) | - Translation of the entire education program from German into 4 other languages: English, Arabic, Russian, and Turkish - Communication of information through films and illustrations (language-independent knowledge transfer) |
| 2 | Health socialization: app users establish an open, prevention-oriented health behavior | - Attend dentist appointments regularly (twice a year; “knowledge” and “action control”) - Take advantage of preventive measures such as professional tooth cleaning (once a year; “action control”) - Act timely when symptoms or pains arise (“action control” and “risk perception”) - Be aware of health care coverage and possibly additional treatment costs (“knowledge” and “awareness”) - Know which specialist to seek out in case of problems (“knowledge”) - Identify patients’ and dentists’ responsibilities (“awareness” and “action control”) | - Information about:   - The German health care system   - Financing and treatment options   - Processes and requirements in dental practices   - Other sources of information and contact points |
| 3 | Oral hygiene habits: app users establish an efficient oral hygiene | - Be aware of their own oral health behaviors (“awareness”) - Know what good oral health behavior is and what techniques (eg, tooth brushing) to adopt (“knowledge”) - Be aware of the risks of dental diseases and their own susceptibility to them (“knowledge” and “risk perception”) - Use appropriate oral hygiene products (“skills”) - Be aware of the impact of inefficient oral hygiene practices on oral health or overall health (“knowledge” and “risk perception”) - Feel confident and positive about their oral health behaviors (“self-efficacy”) | - Education about proper oral hygiene (eg, frequency of tooth brushing, flossing, and dental visits and instruction videos on proper tooth brushing techniques or use of dental floss) - Education about health risks related to poor oral hygiene |
| 4 | Eating habits: app users establish teeth-friendly nutrition | - Know the impact of certain foods on oral health (“knowledge”) - Feel confident to know which food items to avoid for better oral health (“self-efficacy”) | - Education about healthy and unhealthy eating habits - Education about oral health risks related to poor nutrition - Examples of food items from different cultures and countries, in the context of oral health |
